# Boxer’s Knuckle

**DOI:** 10.5334/jbsr.2620

**Published:** 2021-11-22

**Authors:** Nina Watté, Luc Walschot, Filip Vanhoenacker

**Affiliations:** 1 UZ Brussel, BE; 2 AZ Maarten, Mechelen, BE; 3 AZ Sint-Maarten and University (Hospital) Antwerp/Ghent, BE

**Keywords:** boxer’s knuckle, dynamic ultrasound, magnetic resonance imaging, metacarpophalangeal joint

## Abstract

**Teaching Point:** Persistent metacarpophalangeal joint pain after clenched-fist trauma with an unremarkable conventional radiography justifies further examination with dynamic ultrasound for detecting extensor hood injuries.

## Case Presentation

A 45-year old right-dominant man punched a wall with his right clenched fist. Two weeks later, he presented with persistent pain and swelling of the metacarpophalangeal (MCP) joint of the middle finger. At physical examination, a tender swelling at the dorsal side of the MCP joint was observed. Furthermore, a slight radial deviation was noted. Neurovascular examination is normal. Analgesics did not improve pain.

**Figure 1:**
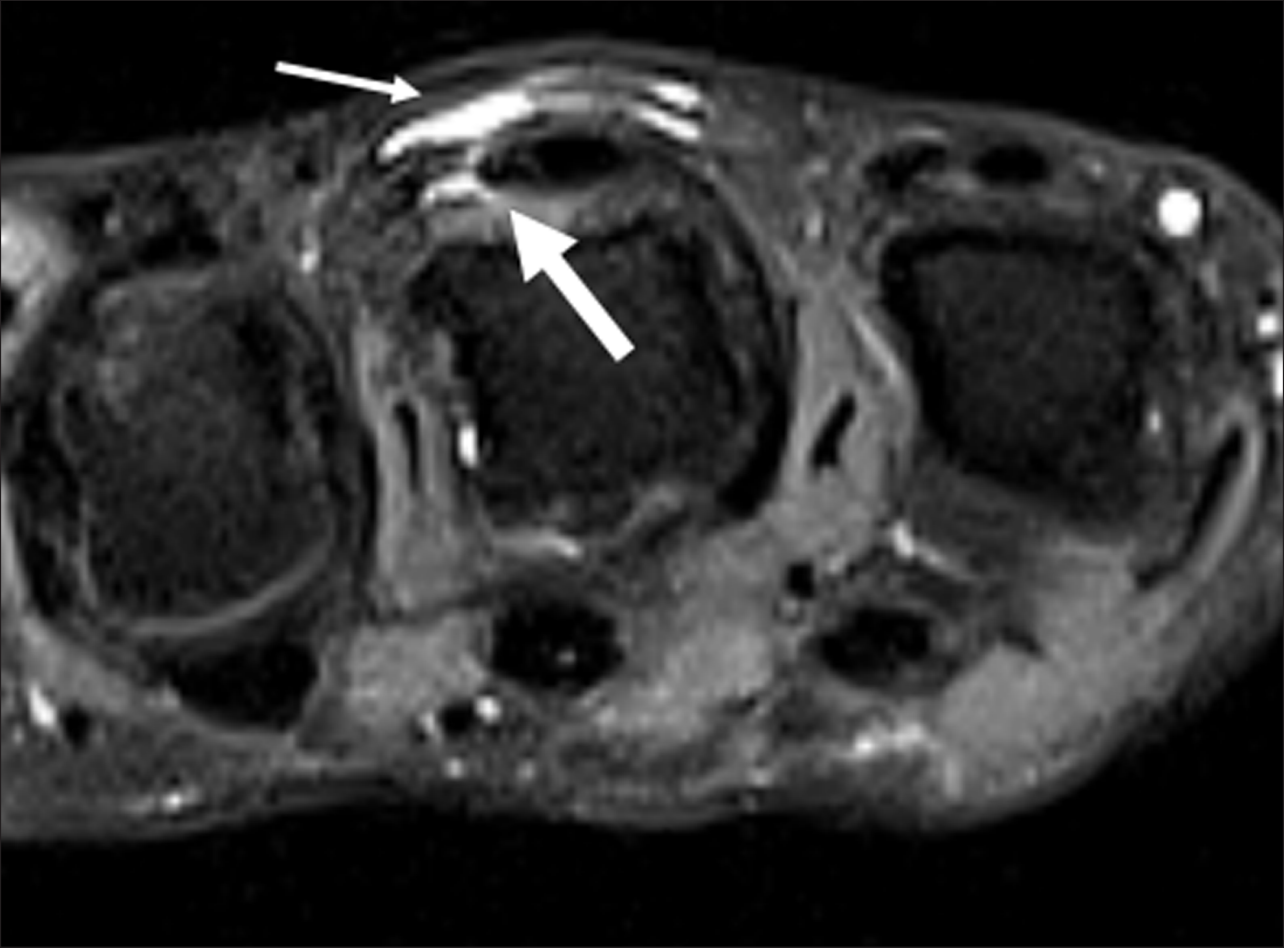


Conventional radiography showed no fracture. Magnetic resonance imaging (MRI) was performed subsequently. Axial fat-suppressed (FS) proton density weighted images (WI) showed a slightly eccentric position of the extensor tendon with relatively radially displacement at MCP of the middle finger. There was focal disruption at the ulnar sagittal band (***Figure 1***, bold arrow) and a thin-walled fluid collection on the dorsal side of the extensor tendon of MCP (***Figure 1***, thin arrow).

**Figure 2:**
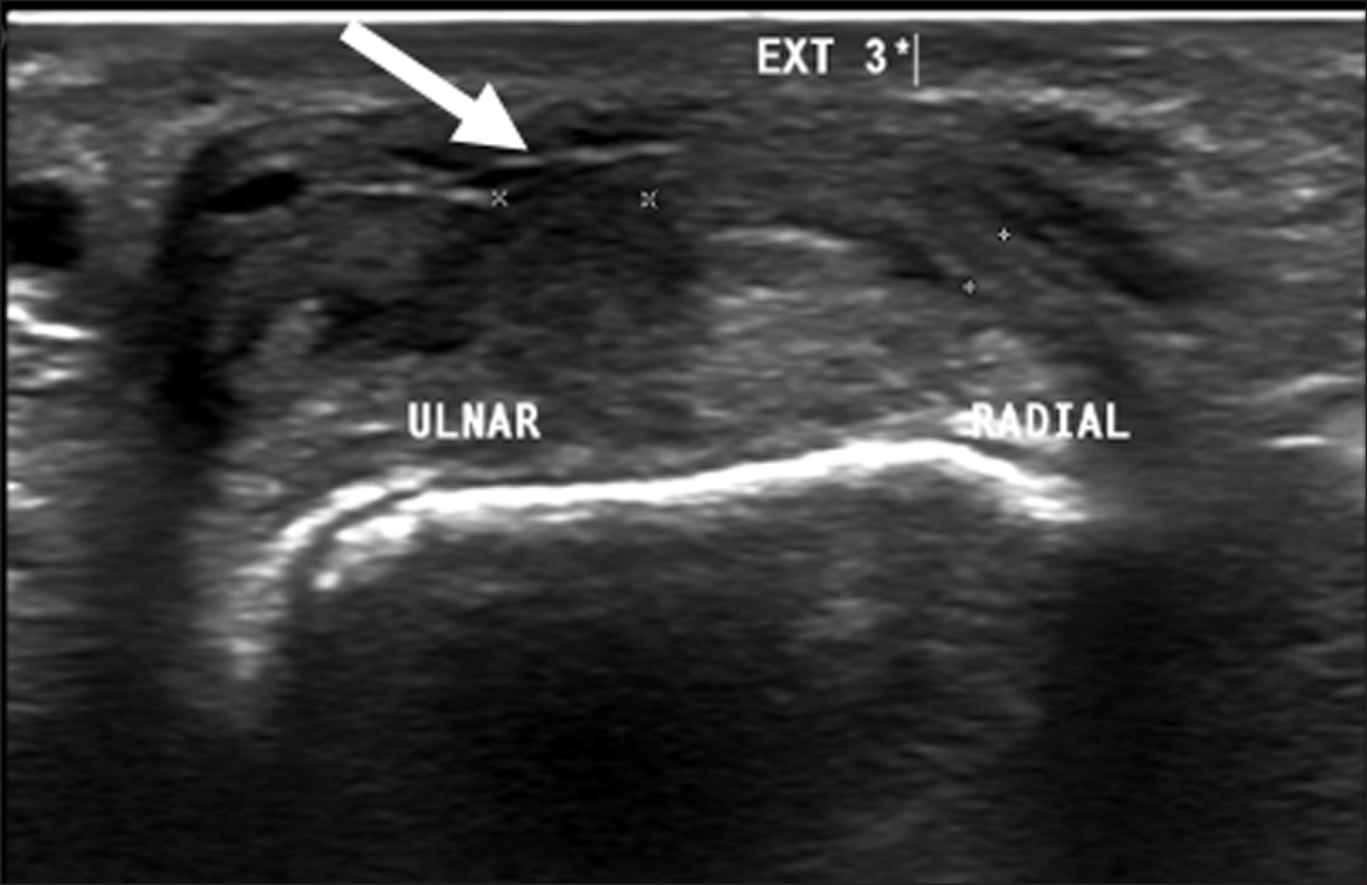


**Figure 3:**
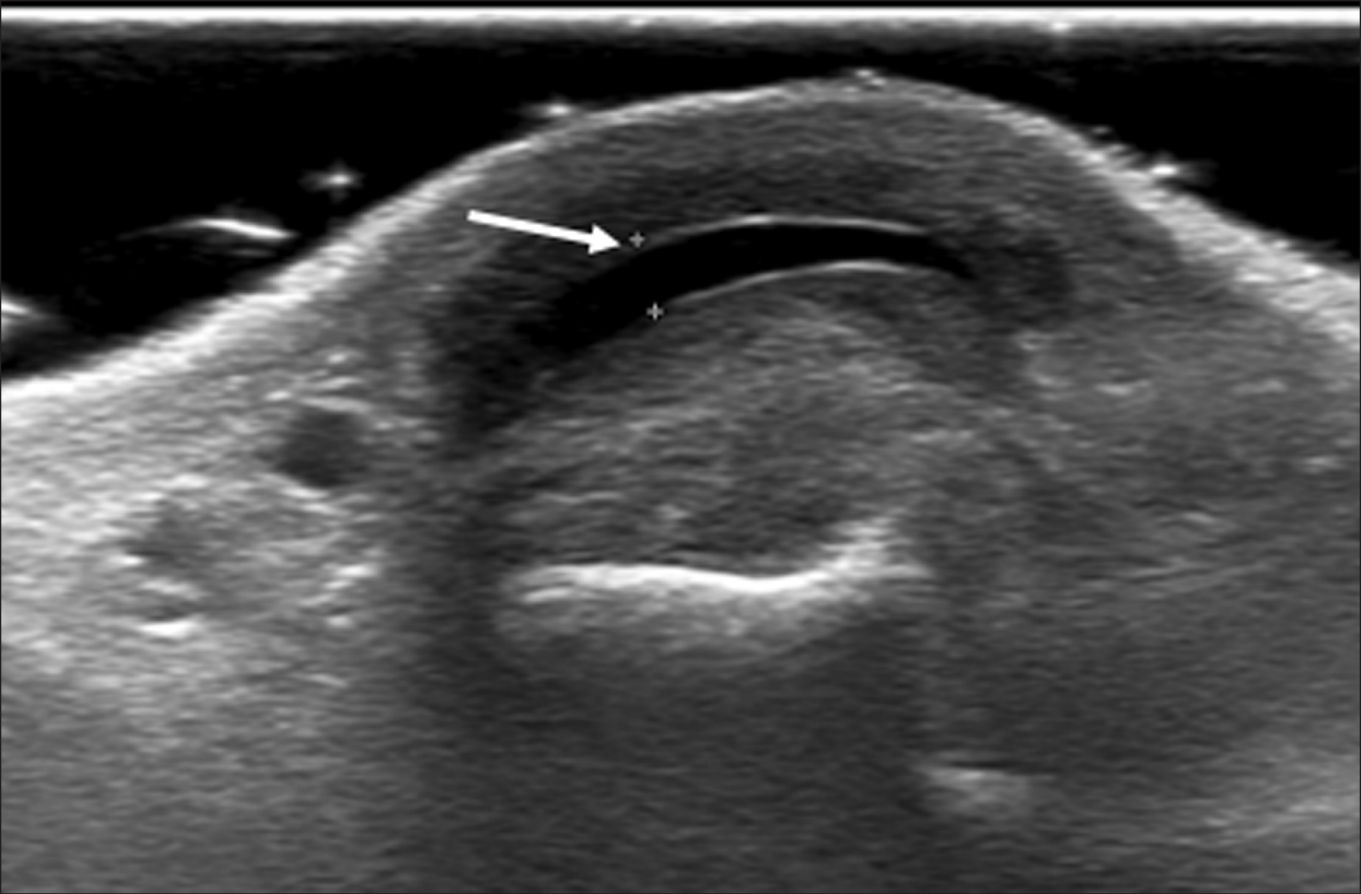


Subsequent dynamic ultrasound was performed. This examination confirms the focal disruption of the ulnar dorsal cap of the extensor tendon (***Figure 2***, bold arrow) leading to radial subluxation with contiguous fluid collection on the dorsal side at the level of the head of the third metacarpal (***Figure 3***, thin arrow).

## Comment

Sagittal band rupture is often referred to as a “Boxer’s knuckle”. This lesion, which is common in boxers, occurs often after clenched-fist trauma and mostly at the third MCP. Clinical findings consist of pain, swelling, incomplete joint extension, and subluxation of the extensor tendon. Sagittal bands are transversely located ligaments, centralizing the extensor tendon. Due to ulnar deviation predisposition of the MCP joint and anatomic weakness, the radial band ruptures more often than ulnar sagittal bands [1]. In our case, however, the ulnar sagittal band was involved. Abnormal signal intensity or echogenicity of the thickened and possible discontinuity of the sagittal band with edema of the dorsal soft tissues suggest the diagnosis on MRI and ultrasound. Dynamic ultrasound (US) can confirm the extensor tendon subluxation during MCP joint flexion, which is an advantage compared to static MRI. Conservative treatment can be considered in acute cases, while surgical repair of the sagittal bands is standard of care in severe acute and chronic ruptures. In our patient, surgical repair was done.
